# Quantifying pediatric patient need for second- and third-line HIV treatment: A tool for decision-making in resource-limited settings

**DOI:** 10.1371/journal.pone.0224226

**Published:** 2019-11-14

**Authors:** Perry Mohammed, Andrea Linden, Maura Reilly

**Affiliations:** 1 Global Public Health, Johnson & Johnson, High Wycombe, Hertfordshire, United Kingdom; 2 Rabin Martin, New York, New York, United States of America; University of Ghana College of Health Sciences, GHANA

## Abstract

As national HIV programs across the world mature and continue to scale up towards UNAIDS’ 90-90-90 targets, it is increasingly important to accurately estimate HIV treatment needs in pediatric patient populations to prepare for anticipated increases in demand. This is particularly vital in sub-Saharan Africa, where the bulk of the global pediatric HIV burden remains concentrated, and for treatment-experienced populations, for which data are severely limited. This article discusses the conceptual framework behind and application of a five-year country-level quantification and decision-making tool aimed at providing national HIV programs and their partners with a better understanding of their evolving national HIV treatment and programming needs for second-and third-line pediatric populations. The conceptual framework of the algorithm which undergirds the tool is the patient pathway, along which key influencing factors that determine whether pediatric HIV patients are linked to care, remain in treatment, and are appropriately switched to later lines of treatment are accounted for quantitatively. Excel-based and arithmetic, the algorithm is designed to use available national, regional, and global data for factors impacting patient estimates including treatment coverage; routine viral load testing; viral load non-suppression; confirmed treatment failure; and patient loss to follow up—outcomes for which data are generally very limited in this patient population. The ultimate output of the tool is an estimate of the aggregate annual number of patients by treatment line. Given the limitations in available data for pediatric HIV, particularly for patients on second- and third-line treatments, this tool may help fill a data gap by providing a mechanism for policymakers to scenario plan, thus aiding resource allocation decisions for pediatric HIV program scale-up. The tool may be used to streamline national antiretroviral procurement of later lines of treatment, especially in resource-limited settings, and may also be used to add value to broader HIV policy and planning processes at the national level.

## Introduction

The state of the global HIV epidemic among children under the age of 14 has changed considerably over the past decade. The scale-up of prevention of mother-to-child transmission programs has contributed to a significant reduction in the number of new HIV infections among children–from 270,000 in 2010 to 160,000 in 2018 [1]–as well as to a notable reduction in acquired immunodeficiency syndrome (AIDS)-related deaths–from 200,000 in 2010 to 100,000 in 2018 [1]. Although the global scale-up of antiretroviral therapy (ART) has also contributed to these reductions, treatment coverage for children living with HIV continues to lag significantly behind adult treatment: in 2016, 62% [1] of adults living with HIV had access to treatment whereas only 52% [1] of children were on treatment. Access to treatment is particularly challenging for children who have failed first- or second-line therapies in resource-limited countries, as viral load and genotypic resistance tests often are not routinely available and third-line treatment may not be provided free of charge in the public sector. [2,3]

As national HIV programs across the world mature and continue to scale up towards UNAIDS’ 90-90-90 targets, it is increasingly important to accurately estimate HIV treatment needs in pediatric patient populations to prepare for anticipated increases in demand. This is particularly vital in sub-Saharan Africa, where the bulk of the global pediatric HIV burden remains concentrated [4] and for treatment-experienced populations, for which data are severely limited. [5,6] For example, a recent World Health Organization (WHO) scoping review of all available evidence on children failing first- and second-line HIV medicines identified only 18 studies total in this population, including one randomized control trial, seven phase II trials, five prospective cohort studies and five retrospective cohort studies. [7] Moreover, neither UNAIDS nor WHO regularly report country-level data or estimates by line of treatment for pediatric patients in their annual global reports or other data and statistics resources. [8,9]

The New Horizons: Advancing Pediatric HIV Collaborative (“New Horizons”), formed by Janssen Pharmaceutical Companies of Johnson & Johnson with expert input from many stakeholders involved in pediatric HIV care, treatment, and prevention, aims to strengthen the public health approach to care and treatment for children living with HIV. Recognizing that a lack of data continues to impair efforts to improve treatment access for children living with HIV failing first- and second-line treatment, New Horizons developed a quantification tool aimed at providing national governments and other relevant partners with a resource to facilitate discussions, decision making, and short-term pediatric HIV program planning targeting this very underserved patient population. The tool is grounded in the primary factors that determine whether pediatric HIV patients (ages 0–14) are linked to care, remain in treatment, and are switched to later lines of treatment.

## Methodology

### Overview and conceptual framework of the tool

Our Excel-based tool was developed using an iterative approach, integrating publicly-available quantitative data with perspectives gained through stakeholder interviews. The arithmetic algorithm underpinning the tool was constructed to generate an annual estimate of pediatric patient need for second- and third-line HIV treatment in a national program over a five-year period (2018–2021) based on manually changeable parameter data across a variety of domains (described in detail below). In the next section, we describe the results generated by application of the tool when using publicly-available data from the Kenyan context.

**Fig 1:**
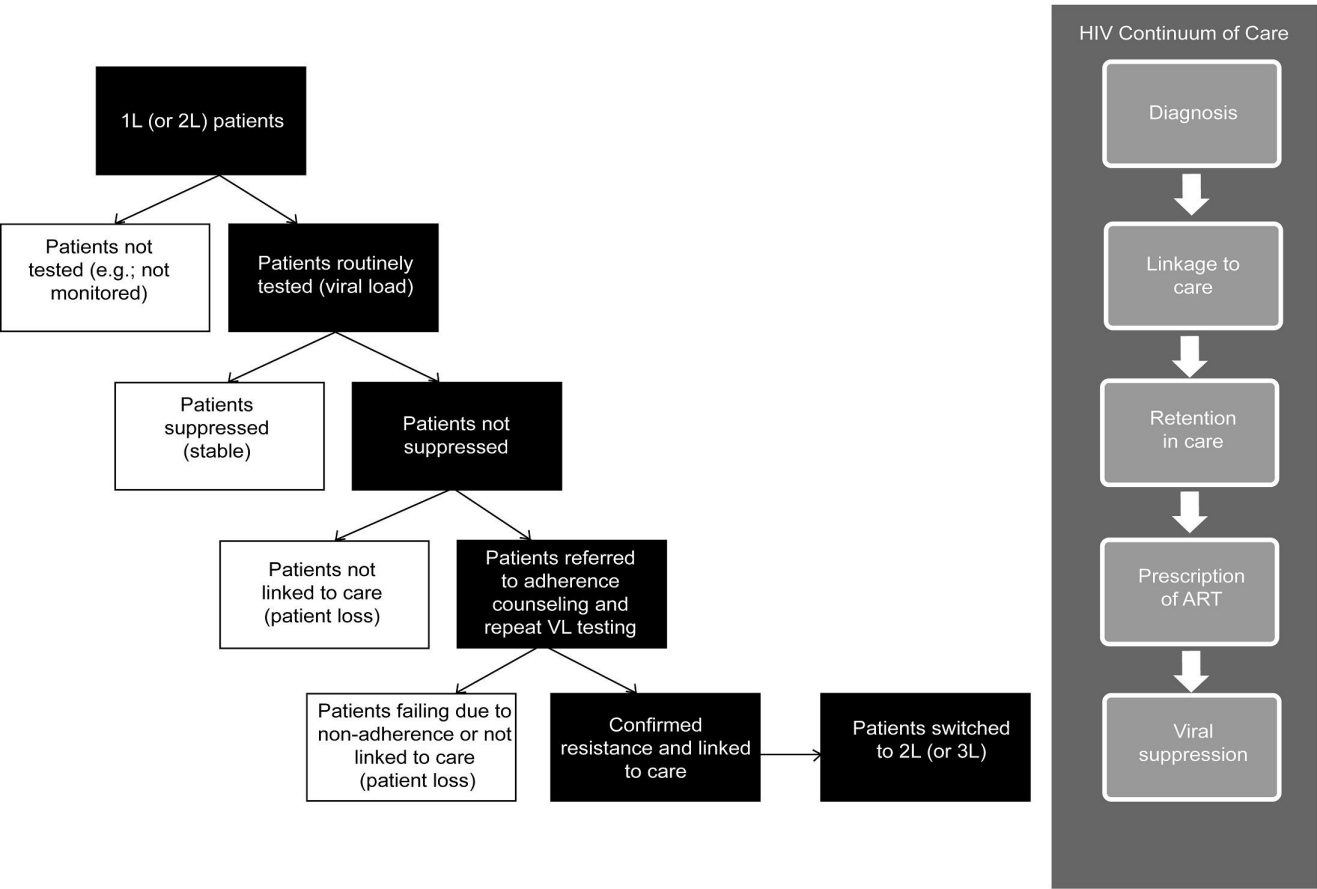
Patient pathway.

The conceptual basis for the tool’s central algorithm is a patient pathway depicting the connections between various key elements that determine whether an HIV-positive child is linked to appropriate care and treatment (Fig 1). The pathway was designed to reflect current evidence [10] and commonly shared understandings among practitioners regarding the pediatric patient journey [11,12], the clinical dimension of treatment failure and switching [13,14], and the on-the-ground realities of HIV treatment in resource-constrained settings [15,16,17,18].

Grounded in treatment guidelines [19,20], the patient pathway served to depict the generalized “steps” that pediatric HIV patients will take when moving to more advanced treatment lines. The sequencing of these steps and patient pool attrition–due to both positive (suppression) or negative (patient loss) factors–at each stage resulted in a pathway in which only a subset of patients progressed. A core assumption that resulted from this approach was that “lost” patients did not re-enter the pathway at a later stage nor did they progress to a later line of treatment.

### Tool parameters

Key parameters of the tool include pediatric HIV treatment coverage; routine viral load testing rate; viral load non-suppression rate; confirmed treatment failure rate; and patient loss. Where appropriate, the tool allows for differences in these rates for first- and second-line treatment to be reflected. Additional foundational parameters, such as mortality, new cases, and rate of aging out, were included in the algorithm as well. Table 1 provides an overview of the parameters, the definitions applied, and methodological notes regarding sources and calculations.

**Table 1:** Overview of model parameters.

Parameter	Description	Notes on Data Sources	Potential Data Sources
*Key Parameters*			
Pediatric HIV treatment coverage	Percentage of eligible children (0–14 ages) in need of ART reached	National-level coverage rates are generally publicly reported.	Data reported by Ministries of Health, UNAIDS, United Nations Children's Fund (UNICEF), WHO
Routine viral load testing (different rates for first- and second-line treatment)	Percentage of ART patients who undergo viral load testing (assumes 1 routine test per patient per year)	Limited data exists regarding routine viral load testing among pediatric patients. In the absence of appropriate national-level data, values should reflect clinical study data, available testing data (adult populations), and qualitative data on testing infrastructure and routine testing practices in resource-limited settings.	Clinical study data, qualitative data from peer-reviewed articles, UNAIDS data (adults only)
Viral load non-suppression rate (different rates for first- and second-line treatment)	Percentage of patients receiving viral load tests who are not virologically suppressed	Limited data exists regarding pediatric viral load suppression rates. In the absence of appropriate national-level data, values should reflect relevant clinical study data, available viral load suppression rate data (adult populations), and qualitative data on factors influencing suppression rates and trends.	Clinical study data, qualitative data from peer-reviewed articles, UNAIDS data (adults only)
Confirmed treatment failure (different rates for first- and second-line treatment)	Percentage of patients whose treatment failure is confirmed by a confirmatory viral load test (or, where available and appropriate, a resistance test) prior to switching to second-line	Limited data exists regarding pediatric HIV treatment failure. In the absence of appropriate national-level data, values should reflect a survey of clinical studies and observations from practicing clinicians. Recent WHO data revealed a broad range of estimated treatment failure rates (from over 30% to 0.5%).	Clinical study data, qualitative data from peer-reviewed articles, WHO data
Patient loss (different rates for first- and second-line treatment)	Percentage of patients lost following viral load testing and not linked to additional care (drivers include lack of access, loss to follow up, and mortality)	At various stages over the course of treatment, patients will be lost due to lack of access (to treatment, services, and/or care), loss to follow up, and mortality. In the absence of appropriate national-level data, values should reflect clinical study data. A review of recent studies suggests that adherence is a larger problem for pediatric patients than for adult patients and that there are greater barriers to access for pediatric HIV services and care as compared to adult services.	Clinical study data, qualitative data from peer-reviewed articles
*Foundational Parameters*			
Mortality	Annual number of pediatric HIV patient deaths expressed as a percentage of the total pediatric HIV patient pool	National-level AIDS-related mortality rates for the pediatric HIV+ population are generally publicly reported.	UNAIDS data (children)
New cases	Decrease in number of new cases of HIV infections among children (0–14 years) expressed as a percentage change	National-level data on new cases of pediatric HIV are generally publicly reported.	UNAIDS data (children)
Rate of aging out	Percentage of children who age out of the 0–14 age cohort (on an annual basis)	In the absence of national-level data for the pediatric HIV+ population, demographic data for the general population within the 0–14 age cohort can be used as a proxy.	UN World Population Prospects

## Results

### Sample country application–Kenya

In Kenya, recent initiatives such as PEPFAR’s Accelerating Children’s HIV/AIDS Treatment Initiative [21,22] have helped to reduce the gap in treatment coverage between adults and children and expand access to treatment for children in need. As the Ministry of Health seeks to capitalize on these gains and works to continue scaling pediatric HIV treatment programs, forecasting demand for treatment, particularly for later lines of treatment, will become more important to ensure appropriate planning and resource allocation. (Table 2 provides an overview of the state of the epidemic today.)

**Table 2:** Snapshot of epidemic.

Pediatric HIV Burden in Kenya: 2018 (UNAIDS) [1,22,23,24]	
Number of children living with HIV (0–14 years)	120,000
New cases	7,600
Treatment coverage (%)*	61%
Number of children receiving treatment	74,344
Deaths due to AIDS	5,200

*Reported treatment coverage rates vary by reporting source. For example, the treatment coverage rate for 2018 was reported as 61% by the UNAIDS AIDSinfo database, whereas the PEPFAR 2017 Kenya Country Operational Plan reported that pediatric treatment coverage was 82% as of April 2017, and the Kenya National AIDS & STI Control Programme (NASCOP) National ACT Dashboard reported that coverage was 54% in May 2016. While the different reporting periods likely play a role in these differences, comparing different sources in the same period also yields similar inconsistencies. For example, while the PEPFAR 2017 Kenya Country Operational Plan reports 82% treatment coverage of April 2017, UNAIDS reports that pediatric treatment coverage in 2017 was 66%.

**Fig 2:**
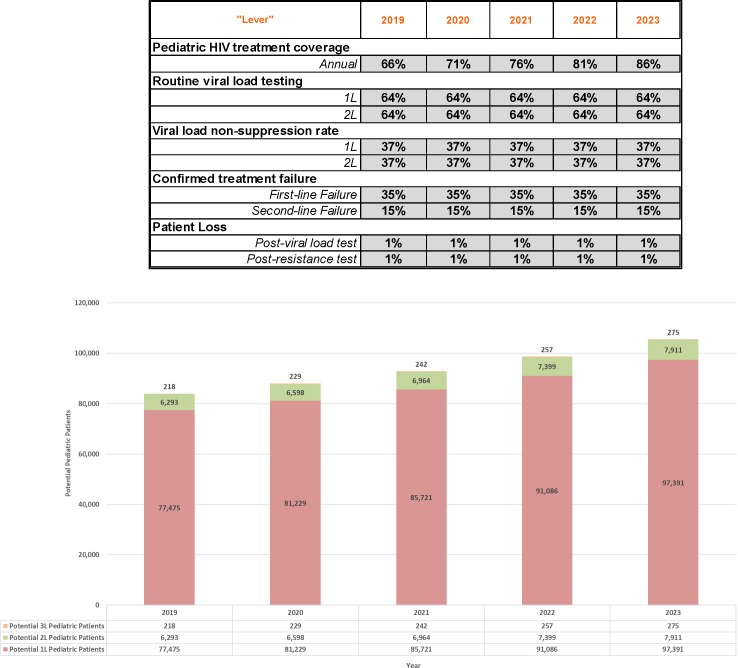
Baseline 2019–2023 forecast.

To generate an estimate of patient need over a five-year period, currently available UNAIDS [1], PEPFAR [22] and NASCOP [23,24] data were used to populate–and calculate or derive as necessary–baseline values for all the model parameters (described above) for 2019–2023. In the absence of reported, country-level data, values were derived based on the following available data points: relevant regional data or estimates; relevant clinical studies; and WHO [25] calculations of the global distribution of patients on ART (see S1 Dataset for additional information on data and calculations). The resulting annual patient estimates (Fig 2) represent a reasonable if somewhat conservative estimate based on publicly available data and latest findings and trends from current country-level programs and initiatives.

**Fig 3:**
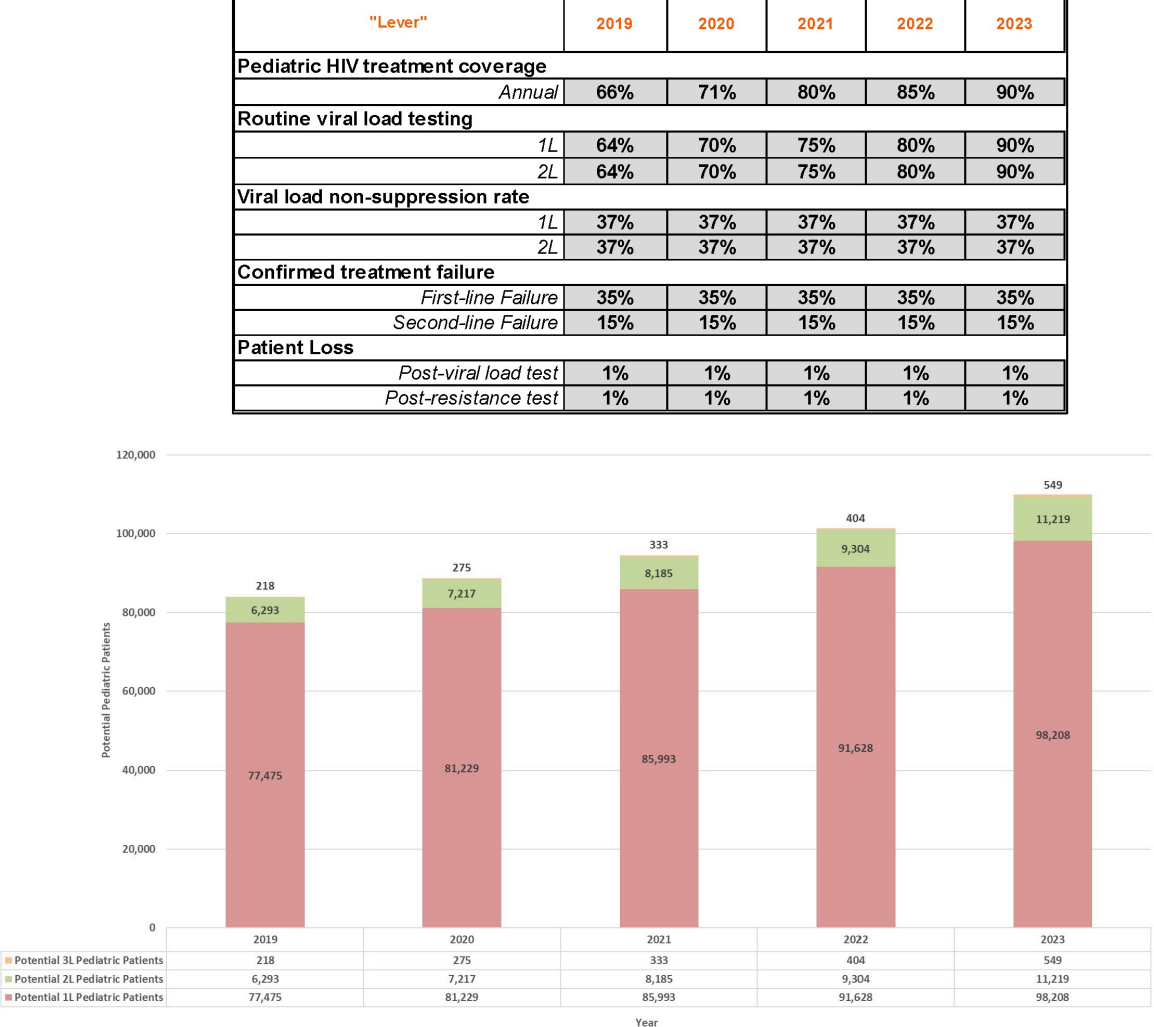
Aggressive growth 2019–2023 forecast.

**Fig 4:**
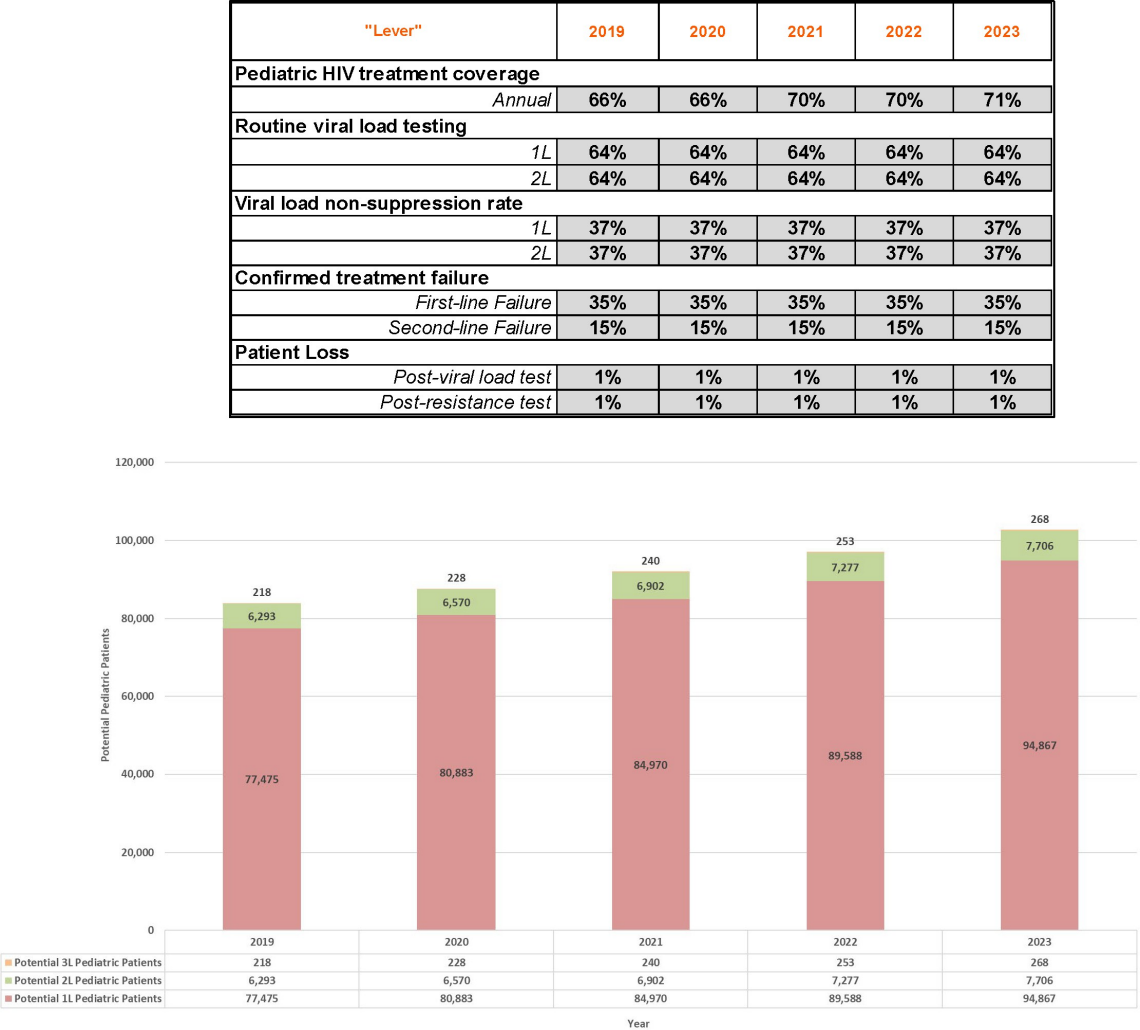
Plateauing growth 2019–2023 forecast.

Using the adjustable levers in the forecasting tool, model parameters can be adjusted to reflect any number of potential scenarios. For example, Fig 3 below depicts an aggressive growth scenario in which 90-90-90 treatment coverage and routine viral load testing goals are achieved by 2023. By contrast, Fig 4 demonstrates what pediatric patient pool growth could look like where treatment coverage growth were slower and if viral load testing rates were to plateau at current estimated levels.

Additionally, as Kenya’s national pediatric HIV treatment program matures and additional resources are dedicated to achieving 90-90-90 targets, more pediatric patients are likely to be identified and put on treatment; these higher volumes may potentially lead to more pediatric patients advancing to later lines of treatment. Model parameters can be adjusted to reflect 90% treatment coverage and 90% viral load testing coverage (assuming a standard of 1 test per year as indicated in treatment guidelines in many resource-limited settings) so as to forecast anticipated patient need for later lines of treatment under these conditions, particularly third-line treatment. The difference between anticipated third-line patient estimates using those parameters and the third-line patient estimates using parameters that reflect the current national treatment landscape represents a way to conceptualize and quantify future pediatric patients in need of such treatment (Table 3). It also offers an approach for conceptualizing potential “unmet need” for third-line treatment by offering a method for estimating the range of potential patients in need of treatment under different scenarios. Comparing these scenarios to anticipated and/or planned national treatment program resource levels could help governments and other relevant partners identify potential shortfalls.

**Table 3:** Anticipated third-line patients under different scenarios.

	2019	2020	2021	2022	2023
**Anticipated total third-line patients with 90% treatment coverage and 90% routing viral load testing**	442	471	501	534	572
**Patients on third-line treatment currently forecasted**	218	229	242	257	275
**Difference between current forecast and anticipated total with partial 90-90-90 achievement**	224	242	259	277	297

## Discussion

### Strengths and limitations of the tool

Designed to be used for quantification and decision making at the national level for a population of patients for which little data—historic or current—exists, the tool’s strength lies in its changeable levers and ability to be used for scenario planning. Notably, the patient pathway algorithm which undergirds the tool was designed to reflect the on-the-ground conditions and patient journey in countries with the highest prevalence of pediatric HIV today where scenario planning would be of greatest use. Additionally, the baseline values used to populate the model parameters–and assumptions used to derive proxies as needed–in our Kenya example were calculated based on recent trends in the pediatric HIV treatment data as well as a synthesis of findings from available literature.

Limited availability of real-world data, however, posed a challenge to the development of the tool and is a limitation of its future application. Age-disaggregated data is not widely available; in some cases, different data sources (e.g., the U.S. President’s Emergency Plan for AIDS Relief [PEPFAR] vs. UNAIDS vs. Ministry of Health) may be reporting different statistics for comparable time periods. The use of adult data as a proxy, which was necessary in our Kenya example, can lead to distortions as adherence, retention, and loss to follow up rates are generally higher among adult patient populations as compared to pediatric patients. The accuracy of available data, in turn, impacts the accuracy of the tool’s output and the resulting patient estimate.

Additionally, as the tool’s output is limited to aggregate annual patient numbers, ART nuances that could impact patient numbers within a given year are not captured (e.g., length of time on treatment prior to failure, age at treatment initiation, etc.). Moreover, the tool’s design does not factor in the possibility of “re-entry” for patients lost at an earlier stage. Although re-entry may happen in practice, current available data is neither robust nor generalizable enough to derive a proxy that could be applied to the tool with a reasonable degree of confidence. Lastly, the algorithm is arithmetic-based, and, as a result, the forecast is limited to linear patient pool growth (within annual intervals as parameters can be adjusted by year included in the time horizon).

### Comparison with other quantification tools

As national HIV programs continue focusing on the challenge of reaching HIV-positive children in need of treatment, patient quantification tools can add value to broader HIV policy and planning processes at the country level. A number of estimation and projection tools are currently available for country-level use, including the SPECTRUM AIDS Impact Module (SPECTRUM AIM) [26] and the Clinton Health Access Initiative Simple Tool [27]. Used by UNAIDS, SPECTRUM AIM is a mathematical model that uses country-level demographic data and HIV surveillance, survey, and program data to generate estimates of HIV indicators (historical and short-term projections). These indicators include key statistics such as the number of people living with HIV, HIV incidence, number of new infections, etc. As country reporting has evolved to meet 90-90-90 requirements, UNAIDS SPECTRUM AIM estimates have expanded to capture progress towards these goals and include indicators such as viral suppression. Many of these indicators are not yet age-disaggregated and forecasts are limited to the immediate year, posing a challenge for pediatric HIV planning. SPECTRUM AIM also does not report statistics broken down by line of treatment.

While the Clinton Health Access Initiative (CHAI) Simple Tool [27] provides a three-year forecast, this tool focuses on quantifying antiretroviral (ARV) needs instead of estimating patients and other key HIV indicators. A morbidity-based commodity forecast, the Simple Tool uses the number of patients per ART regimen treated in the past 12 month period (actual or estimated) and estimated incidence or prevalence rates to generate an estimate of the number of patients that can be expected to be treated and the number of ARVs these patients will need. The pediatric portion of the Simple Tool also incorporates the weight distribution of the pediatric cohort in question as well as weight distribution by ARV formulation. As a quantification tool, the Simple Tool focuses on estimating the supply needs for ARVs and does not incorporate dimensions of patient treatment access that also serve to determine the size of potential pediatric patient pools in practice.

## Conclusion

Among our analytical tool’s potential applications, we envision it being used to complement ongoing commodity forecasting to strengthen and streamline national ARV procurement, particularly for second- and third-line ARVs, as the tool accounts for clinical and patient-specific challenges associated with treatment switching in resource-limited settings. Additionally, the tool could be used to compare a country’s current patient forecast to an anticipated (or desired) scenario–e.g., partial achievement of the 90-90-90 targets. This comparison data would help policymakers and partners think critically and creatively about potential patients in need of treatment that they have either not yet identified or that they will need to account for as they scale up their national programs. Given ongoing pediatric HIV data limitations, particularly for later lines of treatment, a patient estimate of this type can provide a national baseline to ground planning discussions and support strategy development and decision making around resource allocation for effective pediatric HIV programs scale up.

## Supporting information

S1 Dataset Kenya model parameters. (XLS)
